# Effect of a monitored home-based exercise program combined with a behavior change intervention and a smartphone app on walking distances and quality of life in adults with peripheral arterial disease: the WalkingPad randomized clinical trial

**DOI:** 10.3389/fcvm.2023.1272897

**Published:** 2023-11-22

**Authors:** Ivone Silva, Célia Sofia Moreira, Susana Pedras, Rafaela Oliveira, Carlos Veiga, Luís Moreira, Daniel Santarém, Daniel Guedes, Hugo Paredes

**Affiliations:** ^1^ Angiology and Vascular Surgery Department, Centro Hospitalar Universitário de Santo António, Porto, Portugal; ^2^ UMIB—Unit for Multidisciplinary Research in Biomedicine, ICBAS—School of Medicine and Biomedical Sciences, University of Porto, Porto, Portugal; ^3^ ITR—Laboratory for Integrative and Translational Research in Population Health, Porto, Portugal; ^4^ Centre of Mathematics & Faculty of Sciences, University of Porto (CMUP & FCUP), Porto, Portugal; ^5^ Universidade de Trás-os-Montes e Alto Douro, Vila Real, Portugal; ^6^ INESC TEC–Institute for Systems and Computer Engineering, Technology and Science, Porto, Portugal

**Keywords:** home-based exercise therapy, peripheral arterial disease, intermittent claudication, quality of life, smartphone application

## Abstract

**Aims:**

Investigate whether a Home-based Exercise Therapy (HBET) program for patients with Peripheral Arterial Disease (PAD) and Intermittent Claudication (IC) with a behavior change intervention, supported by a smartphone application, is effective in improving walking distances and performance, and quality of life (QoL) over 6 months.

**Methods and results:**

This was a single-center, prospective, two-arm, single-blinded randomized controlled trial including 73 patients with PAD and IC, and three assessment moments: baseline, 3, and 6 months. Participants were randomized to receive a walking exercise prescription, with the support (*n* = 38) or without the support (*n* = 35) of the WalkingPad app, between January 2021 and July 2022. Both groups received two face-to-face behavior change sessions and 12 structured and targeted reinforcement phone calls over 6 months. Primary outcomes were between-group differences in pain-free walking distance (PFWD), functional walking distance (FWD), maximal walking distance (MWD), and 6-min walk distance (6 MWD) at 3 and 6 months. Secondary outcomes were QoL and walking impairment. Seventy-three patients (mean age 64 ± 7.2 years, 88% men) participated in this study, 60 of whom completed the three assessment moments. The whole sample significantly improved all primary outcomes in the first 3 months; that is, the average PFWD (151.1 m), FWD (175.2 m), MWD (171.1 m), and 6 MWD (30.8 m) increased from T1 to T2. Only MWD exhibited a significant average increase (35.0 m). Secondary outcomes also increased from baseline to 3 and 6 months. There were no between-group differences, except for MWD, which showed a greater increase at 6 months in the group that used the app, excluding patients with weak walking ability and extreme anxiety symptoms at baseline.

**Conclusion:**

The intervention improved distances and walking skills as well as the physical, mental, and disease-related quality of life among adults with PAD and IC. The group that used the WalkingPad app improved their MWD in 6 months compared to the control group, except for patients with poor walking ability and extreme anxiety symptoms, which suggests the effectiveness of the WalkingPad app for patients with high walking ability and no severe anxiety symptoms. More research is needed to determine the durability of these findings and to explore what app functionality might promote the other outcomes.

**Clinical Trial Registration:**

https://clinicaltrials.gov (NCT04749732).

## 1. Introduction

Lower extremity peripheral arterial disease (PAD) is associated with reduced walking capacity and increased cardiovascular morbidity and mortality risk (1). Regular exercise significantly improves walking ability and performance in patients with PAD and is the first-line therapeutic measure recommended by clinical practice guidelines (2). Home-based exercise therapy (HBET) is a structured, unsupervised, self-directed walking program performed in the patient's residential area rather than in a clinical setting (3, 4). HBET programs are effective in improving walking performance and physical activity in the short term (3 months), but it is not entirely clear whether they are effective in the long term (6 months) (4, 5). However, adherence to exercise therapy is typically low because of the lack of supervision (5) (unlike supervised exercise therapy) and human nature (people tend to seek pleasure and avoid pain) (6).

Patients with PAD often have a low quality of life (QoL) associated with the inability to walk caused by pain (7). Moreover, there are high prevalence rates of anxiety and depressive disorders among patients with PAD, which in addition to being underdiagnosed (8), the emotional state plays a key role in decreasing disposition and adherence to physical exercise, increasing barriers and consequently restraining improvement of PAD (9).

Developing patients' intrinsic motivation through a theory-based behavior change intervention (10–12) is crucial to promote adherence to exercise therapy and desirable outcomes. In addition, patients with PAD are recommended to receive advice about lifestyle changes and medical therapies to reduce the risk of atherosclerotic complications (13).

Smartphone apps are innovative, low-cost, and proven effective tools for improving walking abilities and distances (14–17). However, current HBET programs use smartphone apps primarily through commercially available fitness apps that sync with wearable activity monitors (WAM) to record and access data. Specific applications for PAD are scarce, and their efficacy is still unclear (18).

We hypothesized that the WalkingPad study (a single-center randomized clinical trial), a home-based exercise program with a behavior change intervention and a smartphone app—WalkingPad—will improve walking ability, distances, and QoL in people with PAD and intermittent claudication (IC) compared to a control group.

## 2. Methods

### 2.1. Subjects

The Research Ethics Committee approved this trial on October 22, 2019 (reference: 069-DEFI/068-CES), and the protocol was registered on the US National Library of Medicine (ClinicalTrials.gov) with the identifier NCT04749732 on February 10, 2021. This is a single-center, prospective, two-arm, single-blinded (to patients) randomized controlled trial (RCT) that enrolled participants between March 2021 and July 2022. Participants provided informed consent. The study protocol is published and available online.

### 2.2. Recruitment

Participants were recruited from the outpatient clinic of the Angiology and Vascular Surgery Department of Centro Hospitalar Universitário de Santo António (CHUSA), Porto, Portugal, between January 2021 and July 2022.

### 2.3. Eligibility criteria

Inclusion criteria for the study were: (1) PAD with IC (Fontaine II or Rutherford 1–3) due to atherosclerotic disease and stable IC for more than 3 months; (2) Ankle Brachial Index (ABI) below 0.9 at rest or below 0.73 after exercise (20% decrease); (3) Age range between 50 and 80 years; (4) MWD in treadmill test between 50 and 600 m.

Exclusion criteria were: (1) asymptomatic PAD; (2) critical ischemia (Fontaine III/IV or Rutherford 4–6); (3) previous lower extremity vascular surgery, angioplasty, or lumbar sympathectomy; (4) any condition other than PAD that limits walking; (5) unstable angina or myocardial infarction diagnosed in the last 6 months; (6) inability to obtain ABI measure due to non-compressible vessels; (7) use of cilostazol and pentoxifylline initiated within 3 months before the investigation; (8) active cancer, renal disease, or liver disease; (9) severe chronic obstructive pulmonary disease (GOLD stage III/IV); (10) severe congestive heart failure (NYHA class III/IV); (11) diagnosis of a psychiatric disease that impairs daily life activities and/or with medical records of decompensation episodes in the last year and/or non-adherence to drug therapy; and (12) cognitive impairment (MMSE ≤ 15 for illiterate patients, 22 for those with 1–11 years of schooling; 27 for >11 years).

### 2.4. Randomization and masking

Participants were randomly assigned in a 1:1 ratio to receive an exercise prescription with a behavior change intervention supported or not by a smartphone app, using a computer-generated randomization system, with randomly selected block sizes of 5 stratified by age (50 to 65 years old; 66 to 80 years old), and maximal walking distance (50 to 325 m; 326 to 600 m) (flow diagram as Supplementary Material S1). It was impossible to mask participants, the outcome assessor, and the psychologist for group allocation after randomization due to the nature of the interventions. The statistician was masked for group allocation.

### 2.5. Interventions

All participants received standard PAD treatment, a physical exercise prescription, and a behavior change intervention. Standard treatment for PAD and IC followed the guidelines of the American College of Cardiology/American Heart Association Task Force on Clinical Practice Guidelines (AHA/ACC) (2, 3, 19, 20).

The physical exercise prescription consisted of walking sessions, performed for at least 30 min per session, three times a week in the area of residence of the participants. Near-maximal pain during training was the outcome of claudication pain (21). The behavior change intervention was based on Self-Determination Theory (SDT), which focuses on the type and quality of motivation, and the satisfaction of basic psychological needs, as pre-eminent behavioral determinants (22–24). According to this theory, individuals become more autonomous or self-determined on a continuum that ranges from external motivation to internalization of motivation. As this continuum progresses, individuals are more likely to engage in new, long-term behaviors. To achieve this, motives or extrinsic reasons for changing and adhering to a new behavior must be internalized and become as intrinsic as possible. Thus, the behavioral change intervention aimed to facilitate this internalization process to nurture a more autonomous motivation. Also, motivational interviewing principles are considered effective in promoting and encouraging change (11, 25, 26). Patients received two face-to-face behavior change sessions, at baseline (T1) and 3 months (T2), conducted by a health psychologist, for a total of 4 h (120 min per session). Complementary, booster phone calls over the 24-week intervention period were used to support participants and identify barriers to goal completion, based on a personalized and individualized self-management approach to promote long-term adherence to the exercise prescription (27, 28).

#### 2.5.1. Control group (CG)

Participants assigned to this group received a self-fulfilling walking practice diary to monitor and record walking sessions: frequency (date) and duration of the walking session, the number of stops, and the level of well-being (ranging from 0 very bad to 10 very good) to be filled in each walking session.

#### 2.5.2. Experimental group (EG)

Participants assigned to this group received a smartphone (Samsung Galaxy A41 SM-A415F/DSN, Vietnam) with the WalkingPad app installed. The application has several features that make it an asset to HBET programs: (1) allows adherence to be measured objectively and guides the intervention of health professionals; (2) encourages patient self-management and empowerment—promoting responsible and informed self-management of the disease, and (3) regularly and routinely accompanies the patient during exercise sessions as an “exercise buddy”—providing emotional support. Patients received appropriate coaching on how to use the app and record their data. A web platform (WalkingPad web platform) received data from the app, allowing collaboration between different actors (health professionals, researchers, and engineers) in monitoring patient adherence, promoting patient responsibility in their strategy of care, and adjusting the provision of personalized feedback during booster phone calls to overcome personal barriers to physical exercise. Furthermore, although patients left the hospital with the phone and the application installed, they took with them an Application Installation Guide (Supplementary Material S2) and an Instruction Manual for using the application with a technical support number (Supplementary Material S3).

## 3. Outcome measurements

### 3.1. Medical history and demographics

Medical history and clinical data were collected from the participants' electronic clinical medical records, and sociodemographic data were collected through a clinical interview.

### 3.2. Screening measures

Depressive symptoms were assessed by the Geriatric Depression Scale-5 (GDS), which includes five dichotomous items (yes/no) with higher results corresponding to more depressive symptoms (29, 30).

Anxiety symptoms were assessed by the Geriatric Anxiety Inventory-Short Form (GAI-SF), which includes five dichotomous items (yes/no), with higher results corresponding to more anxiety symptoms (31, 32).

### 3.3. Treadmill evaluation

The modified Gardner-Skinner Treadmill Protocol was used, according to which participants began to walk on the treadmill at 1 km/h with a 0% grade (33). After 2 min, the speed increases to 1.6 km/h at 0% grade. Then the speed is increased by 0.8 km/h every 2 min until reaching 3.2 km/h. After reaching 3.2 km/h, the speed is kept constant, and the grade increases by 2% every 2 min.

### 3.4. 6-Minute walk test

The 6-Minute Walk Test (6 MWT) was performed to evaluate the functional capacity of the individual to walk over a total of 6 min on a 100 ft (≈30 m) hallway (34) according to the American Thoracic Society guidelines (35).

### 3.5. Primary outcomes

The primary outcomes were pain-free walking distance (PFWD), functional walking distance (FWD), maximal walking distance (MWD), and 6 MWD at 3 and 6 months, measured by two tests. The treadmill test (33) and the 6-Minute Walk Test (34, 35). The walking distance measured with a standardized treadmill test is a widely used tool for functional assessment and monitoring of exercise rehabilitation, where PFWD, FWD, and MWD are objective measures of improvement. In turn, the 6 MWT is an individualized test that assesses the submaximal level of functional capacity (35, 36). The patient chooses the intensity of the exercise and can rest during the test, which better reflects the functional level of exercise practiced in daily physical activities. The primary outcomes reflect the effectiveness (beneficial effect) and not the harm (adverse effect) of the intervention.

### 3.6. Secondary outcomes measures

Secondary outcomes were: (1) health-related quality of life (QoL) assessed by the *Vascular Disease-Specific Quality of Life Questionnaire* (VascuQoL-6, scores ranging from 6 to 24, higher scores indicate better QoL) (36–38); and by the 12-Item Short Form Health Survey (SF-12, includes two summary component scores, higher scores indicate better physical and mental QoL) (39, 40); and (2) the *Walking Impairment Questionnaire* (WIQ) was used to assess the daily walking ability of patients in three domains: distance, speed, and climbing stairs (values range from 0 to 100%, and higher scores indicate less impairment in walking abilities/performance) (41).

In most RCTs, especially in clinical settings, it is possible to assess the effectiveness of an intervention using a single primary outcome. However, in many situations, a comprehensive understanding of the effects of an intervention requires analysing multiple outcomes. Indeed, patients' health status cannot be fully evaluated using a single outcome, and distinct outcomes may provide different but equally important information about the effectiveness of the intervention (42). Fortunately, developments in statistical methods have allowed researchers to account for multiple outcomes in RCTs (43), especially those that represent the same outcome at different time points, as well as to use suitable families of distributions. In this study, we expected the WalkingPad intervention to improve patients' MWD, PFWD, FWD, and 6 MWD (42, 43). For this reason, these four outcomes were used to define the effectiveness of the intervention and thus, this study comprises these four primary outcomes.

## 4. Adverse events

As an investigative outcome, the outcome assessor collected adverse events at the 3- and 6 months follow-ups.

## 5. Sample size

When designing this study, we planned to enroll three arms (two intervention groups and one control group) and a total sample of 200 participants, accounting for 20% dropouts and adverse events. However, later, the power analysis carried out indicated that 57 participants were needed for each group and was published in the protocol. However, as it was not possible to recruit this number of participants, due to the Covid-19 pandemic, a new analysis was carried out (at T0) (a posteriori) to find out what effect size the sample would be able to “capture”, that is, whether this number was sufficient to detect between-group effects. Thus, power analysis was performed using the R package WebPower (44). As we were planning to analyze data through mixed-effect models, we selected a repeated-measures ANOVA, which accounts for both within and between-subject effects (45), two groups, three measurements, 5% of significance level, 80% of power, and a 10% attrition rate, grounded in other studies with this population. These conditions provided a minimum Cohen's *f* = 0.36 [or Cohen's *d* = 0.72, in this specific case] (46), which means that it would be possible to find large effect sizes. Therefore, we decided to proceed with the study.

## 6. Statistical analysis

Statistical analyses were conducted using R software, version 4.2.1, and the packages: lme4 (47), lmerTest (48), glmmTMB (49), and interactions (50). The level of significance was set at 0.05.

Analyses regarding the sociodemographic and clinical characterization of the sample and the evolution of the main variables of interest over time were conducted using mathematical models with appropriate families of distribution according to each outcome (dependent variable). Mixed-effects models have been chosen to perform all analyses due to their flexibility and efficiency in analyzing repeated measures, accounting for baseline differences in the outcomes (51). Remarkably, these models yield more efficient estimates, shifting estimates toward each other and making comparisons more conservative (52), ruling out the need to adjust for multiple comparisons or perform post-hoc tests (as with classical procedures such as ANOVA).

To assess differences in the outcomes, mathematical modeling was performed. Before running the models, a suitable family of distributions was selected for each outcome. More precisely, four different types of models were performed: normal linear models for data associated with normality (evaluated using histograms, boxplots, and QQ-plots), logistic models for dichotomous data, COM-Poisson models for count data, and beta models for limited data. Limited data included truncated data, such as MWD, and were previously transformed into percentages. In particular, we draw attention to the fact that, contrary to the majority of similar research works, we did not use the normal distribution to model the MWD since the respective data were subject to truncation and yielded some boundary observations (the normal distribution does not respect bounds). Models were only used to assess differences in outcomes, so the interpretation of model coefficients is not emphasized.

## 7. Results

### 7.1. Sociodemographic and clinical characterization of the sample and between-group differences at the baseline

**Figure 1:**
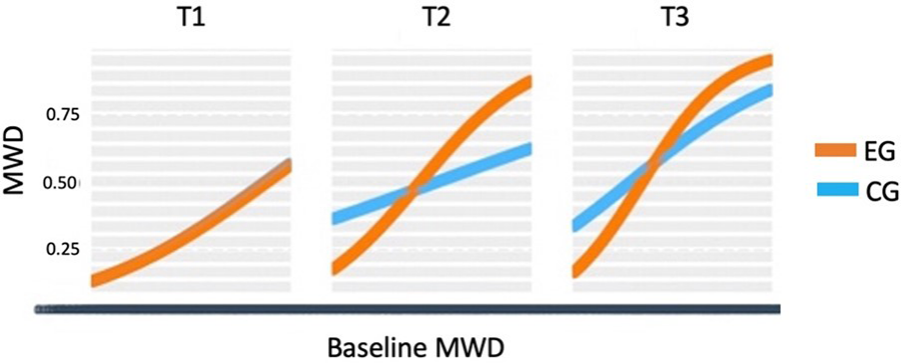
Graphic representation of the between-group differences in the average MWD over time (fitted data), at each time point.

After screening the clinical medical records of patients attending the outpatient clinic of the Angiology and Vascular Surgery Department, 268 patients were identified. Of these, 119 were included and invited to attend a screening evaluation at the hospital to confirm inclusion criteria (at baseline), and 73 were included in the study, randomized, and allocated to one of the two groups (Figure 1). Of these, 60 participated in the three assessment moments. Patients were randomized into two groups: The Control Group (CG)—35 patients (31 men; mean age 64.0 ± 7.16 years) and the Experimental Group (EG)—38 patients (33 men; mean age 63.3 ± 6.73 years). Table 1 shows the sociodemographic and clinical characterization of the sample and between-group differences at the baseline.

**Table 1:** Sociodemographic and clinical characterization of the sample and between-group differences at the baseline.

		Total Sample (*N* = 73)	CG (*N* = 35)	EG (*N* = 38)		
Dichotomous variables	Level 0 vs. Level 1	*n* (%)	*n* (%)	*n* (%)	Logistic models	
					Estimate	*p*
Sociodemographic data						
Sex	Male vs. Female	64 (87.7)	31 (88.6)	33 (86.8)	0.16	0.822
Professional profile	Active/unemployed vs. Retired	25 (34.2)	11 (31.4)	14 (36.8)	−0.24	0.627
Marital status	Married vs. Other^a^	55 (75.3)	26 (74.3)	29 (76.3)	−0.11	0.841
Clinical data						
Hypertension	No vs. Yes	9 (12.3)	4 (11.4)	5 (13.2)	−0.16	0.822
Cholesterol	No vs. Yes	10 (13.7)	5 (14.3)	5 (13.2)	0.10	0.889
Obesity (BMI > 30)	No vs. Yes	53 (72.6)	28 (80.0)	25 (65.8)	0.73	0.178
Diabetes Mellitus type 2	No vs. Yes	40 (54.8)	18 (51.4)	22 (57.9)	−0.26	0.579
Cerebrovascular disease (Str,TIA)	No vs. Yes	63 (86.3)	30 (85.7)	33 (86.8)	−0.10	0.889
Ischemic heart disease	No vs. Yes	52 (71.2)	26 (74.3)	26 (68.4)	0.29	0.581
COPD	No vs. Yes	58 (79.5)	31 (88.6)	27 (71.1)	1.15	**0.073^**
Heart failure	No vs. Yes	70 (95.9)	33 (94.3)	37 (97.4)	−0.81	0.518
Osteoarticular disease	No vs. Yes	52 (71.2)	25 (71.4)	27 (71.1)	0.02	0.972
Liver disease	No vs. Yes	69 (94.5)	34 (97.1)	35 (92.1)	1.07	0.365
Extreme anxiety (GAI-SF = 5)	No vs. Yes	47 (64.4)	24 (68.6)	23 (60.5)	0.35	0.474
Medication						
Acetylsalicylic Acid (ASA)	No vs. Yes	18 (24.7)	4 (11.4)	14 (36.8)	−1.51	0.016*
Clopidogrel	No vs. Yes	58 (79.5)	32 (91.4)	26 (68.4)	1.59	0.022*
Statins	No vs. Yes	6 (8.2)	1 (2.9)	5 (13.2)	−1.64	0.144
Pentoxifylline	No vs. Yes	60 (82.2)	28 (80.0)	32 (84.2)	−0.29	0.639
Insulin	No vs. Yes	65 (89.0)	32 (91.4)	33 (86.8)	0.48	0.534
Cilostazol	No vs. Yes	52 (71.2)	20 (57.1)	32 (84.2)	−1.39	0.013*
NOACs	No vs. Yes	65 (89.0)	31 (88.6)	34 (89.5)	−0.09	0.902
Warfarin	No vs. Yes	69 (94.5)	34 (97.1)	35 (92.1)	1.07	0.365
Oral antidiabetic agents	No vs. Yes	40 (54.8)	18 (51.4)	22 (57.9)	−0.26	0.579
Beta-blockers	No vs. Yes	53 (72.6)	26 (74.3)	27 (71.1)	0.16	0.757
Antihypertensive agents	No vs. Yes	19 (26.0)	8 (22.9)	11 (28.9)	−0.32	0.554
Lifestyle						
Drinking history	No vs. Yes	18 (24.7)	10 (28.6)	8 (21.1)	0.41	0.458
Smoking history	Active smoker vs. Non-smoker	33 (45.2)	14 (40.0)	19 (50.0)	−0.41	0.392
Continuous variables	Min—Max	M (SD)	M (SD)	M (SD)	Regression models	
					Estimate	*p*
Age (years)	50—80	64.0 (7.16)	64.9 (7.61)	63.3 (6.73)	−1.63	0.326
Education (years)	0—21	6.6 (3.95)	6.2 (4.15)	7.0 (3.78)	0.11	0.387
Medications (number)	2—7	4.1 (1.27)	4.3 (1.18)	3.9 (1.32)	−0.11	0.127
Ankle-brachial index (ABI)						
Right ABI pre-exercise	0.35—1.09	0.71 (0.19)	0.72 (0.20)	0.69 (0.19)	−0.03	0.537
Right ABI post-exercise	0.21—1.21	0.64 (0.23)	0.64 (0.25)	0.65 (0.21)	0.002	0.968
Left ABI pre-exercise	0.30—1.23	0.70 (0.18)	0.72 (0.19)	0.67 (0.16)	−0.05	0.191
Left ABI post-exercise	0.20—1.17	0.62 (0.22)	0.63 (0.24)	0.61 (0.21)	−0.02	0.697
Smoking (years)	20—60	44.8 (8.00)	45.9 (7.13)	44.1 (8.70)	−1.81	0.513
Number of diagnoses	2—8	4.6 (1.45)	4.3 (1.30)	4.8 (1.57)	0.09	0.209

^a^ Other = Common-law marriage, widowed, single, or divorced. For dichotomous variables, logistic regression was conducted. For continuous variables, COM-Poisson models have been used in the following cases: education, medications, and diagnosis; all the other cases used normal distribution. Significance: ^*p* < 0.1, *p < 0.05. Significant (or marginally significant) values are highlighted in bold.

### 7.2. Differences in primary and secondary outcomes for the total sample over time

There was a significant main effect of time in primary and secondary outcomes. Results of the mixed-effects models show that the average MWD improved significantly over time for the whole sample (from T1 to T2 and from T2 to T3). The average PFWD and FWD increased over time, but from T2 to T3, the increase was insignificant. The same was true for 6 MWD.

Regarding secondary outcomes, the average QoL disease-related increased significantly over time, from T1 to T2 and from T2 to T3. The average physical and mental QoL increased over time, but from T2 to T3, the increase was not significant. The average WIQ distance increased over time, but from T2 to T3, the increase was only borderline significant. The average WIQ speed increased over time, but from T2 to T3, the increase was not significant. The average WIQ stairs increased from T1 to T2 and T2 to T3 over time. Results are shown in Table 2.

**Table 2:** Differences in the main variables of interest for the total sample, over time.

Variable		T1 (*n* = 73)	T2 (*n* = 68)	T3 (*n* = 60)	T2–T1 (*n* = 68)	T3–T2 (*n* = 60)	T3–T1 (*n* = 60)	Graphic representation
Anxiety	Mean (SD)	3.1 (1.81)	2.9 (1.73)	2.2 (1.89)	−0.3 (1.82)	−0.7 (1.41)	−1.0 (1.89)	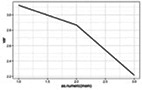
	β				−0.10	**−0.26**	**−0.36**	
	*p*				0.261	0.008**	<.001***	
Depression	Mean (SD)	1.4 (1.40)	1.3 (1.37)	0.9 (1.36)	−0.1 (1.36)	−0.3 (1.40)	−0.5 (1.78)	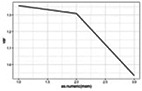
	β				−0.04	**−0.33**	**−0.38**	
	*p*				0.771	**0.053^**	0.027*	
QoL								
Disease-related	Mean (SD)	15.7 (3.05)	17.4 (2.71)	19.3 (3.65)	1.8 (3.69)	1.9 (3.06)	3.7 (4.2)	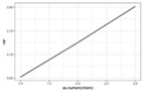
	β				**0.32**	**0.72**	**1.04**	
	*p*				0.003**	<.001***	<.001***	
Physical	Mean (SD)	2.8 (0.84)	3.2 (0.73)	3.3 (0.86)	0.4 (0.90)	0.1 (0.76)	0.5 (0.90)	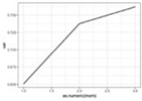
	β				**0.41**	0.14	**0.55**	
	*p*				<.001***	0.242	<.001***	
Mental	Mean (SD)	3.9 (0.90)	4.2 (0.74)	4.4 (0.85)	0.3 (1.01)	0.01 (0.88)	0.3 (1.07)	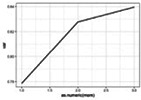
	β				**0.27**	0.07	**0.34**	
	*p*				**0.064^**	0.647	0.026*	
WIQ								
WIQ distance	Mean (SD)	28.8 (26.72)	69.0 (31.79)	70.4 (35.57)	39.1 (37.97)	−3.3 (31.62)	41.5 (35.23)	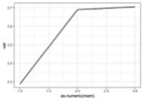
	β				**1.41**	**0.38**	**1.79**	
	*p*				<.001***	**0.065^**	<.001***	
WIQ speed	Mean (SD)	20.8 (13.56)	38.7 (18.16)	40.7 (25.68)	17.6 (20.11)	−0.3 (25.02)	20.1 (26.08)	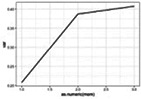
	β				**0.89**	0.21	**1.10**	
	*p*				<.001***	0.191	<.001***	
WIQ stairs	Mean (SD)	40.4 (32.97)	58.0 (37.01)	71.2 (34.33)	16.9 (50.76)	10.6 (40.27)	29.9 (45.24)	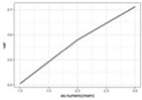
	β				**0.86**	**0.48**	**1.34**	
	*p*				<.001***	0.035*	<.001***	
Treadmill walking								
PFWD	Mean (SD)	122.1 (93.40)	273.6 (160.28)	307.9 (202.16)	151.1 (146.87)	27.1 (152.65)	183.6 (185.46)	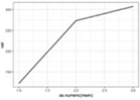
	β				**151.34**	31.50	**182.84**	
	*p*				<.001***	0.132	<.001***	
FWD	Mean (SD)	188.6 (120.97)	363.7 (174.32)	399.6 (210.97)	175.2 (157.44)	24.2 (158.70)	206.6 (173.34)	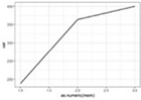
	β				**175.17**	30.26	**205.43**	
	*p*				<.001***	0.154	<.001***	
MWD	Mean (SD)	301.0 (139.23)	478.4 (227.37)	540.0 (277.37)	177.1 (192.52)	35.0 (171.60)	228.5 (222.08)	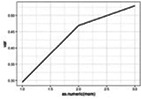
	β				**0.84**	**0.51**	**1.35**	
	*p*				<.001***	<.001***	<.001***	
6-minute walking								
6MWD	Mean (SD)	329.5 (77.95)	363.6 (78.52)	378.5 (78.40)	30.8 (58.69)	7.9 (53.87)	42.8 (61.21)	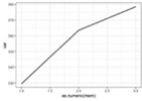
	β				**31.72**	10.74	**42.46**	
	*p*				001***	0.147	<.001***	
6MPM	Mean (SD)	2.5 (0.91)	3.0 (1.13)	3.3 (1.25)	0.7 (1.24)	0.2 (1.36)	0.8 (1.14)	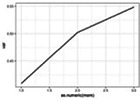
	β				**0.43**	0.04	**0.47**	
	*p*				<.001***	0.750	<.001***	
6MStop	Mean (SD)	1.0 (1.24)	0.6 (0.70)	0.4 (0.83)	−0.5 (1.29)	−0.1 (0.74)	−0.6 (1.17)	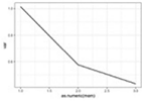
	β.				**−0.59**	−0.20	**−0.79**	
	*p*				<.001***	0.380	<.001***	

Means and graphs obtained with raw data; β is the model estimate and *p* is the *p*-value provided by the corresponding regression model. PFWD: Pain-free treadmill walking distance (in meters); FWD: Functional treadmill walking distance (in meters); MWD: Maximal treadmill walking distance (in meters); 6 MWD: 6-Minute walk distance (in meters); 6MPM: 6-Minute pain distance (in minutes); 6MStop – 6-Minute number of stops. Significance: ^*p* < 0.1, *p < 0.05, **p < 0.01, ***p < 0.001. Number of participants (n) may differ in some rows. Significant (or marginally significant) values are highlighted in bold.

### 7.3. Between-group differences in primary and secondary outcomes, over time

By the end of the study, no significant between-group differences regarding the average of the primary outcomes were found. Results are shown in Table 3. Notice, however, that the sample size of this study only allowed to detection of large effect sizes, which means that increasing the sample size would probably allow uncovering of some medium or small effects.

**Table 3:** Between-group differences in the main variables of interest, over time.

Variable		T1 (*n* = 73)			T2 (*n* = 68)			T3 (*n* = 60)			Within-group change						Graphic representation
											T2–T1		T3–T2		T3–T1		
		CG *n* = 35	EG *n* = 38	EG-CG *n* = 73	CG *n* = 34	EG *n* = 34	EG-CG	CG *n* = 30	EG *n* = 30	EG-CG	CG *n* = 34	EG *n* = 34	CG *n* = 30	EG *n* = 30	CG *n* = 30	EG *n* = 30	
Anxiety	Mean	3.0	3.2		2.9	2.8		2.1	2.3		−0.1	−0.6	−0.9	−0.5	−0.9	−1.1	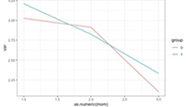
	SD	1.8	1.8		1.8	1.7		1.9	1.9		1.9	1.7	1.3	1.6	2.0	1.8	
	β			0.07			−0.06			0.07	−0.03	0.16	**−0.33**	**−0.20**	**−0.36**	**−0.35**	
	*p*			0.700			0.760			0.715	0.781	0.193	0.021*	0.152	0.010*	0.008**	
Depression	Mean	1.8	1.0		1.3	1.4		0.7	1.2		−0.5	0.4	−0.6	−0.1	−1.1	0.1	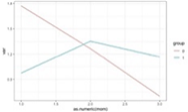
	SD	1.6	1.1		1.2	1.6		1.1	1.6		1.3	1.3	1.3	1.5	1.7	1.7	
	β			**−0.65**			0.02			0.46	**−0.34**	0.33	**−0.58**	−0.14	**−0.92**	0.19	
	*p*			0.019*			0.951			0.167	**0.072^**	0.118	0.022*	0.510	<.001***	0.404	
QoL																	
Disease-related	Mean	16.4	15		17.4	17.4		19.5	19.0		1.1	2.4	2.1	1.6	3.3	4.0	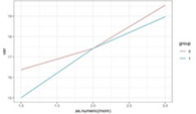
	SD	3.1	2.9		2.8	2.7		3.2	4.1		3.5	3.8	3.0	3.2	4.0	4.5	
	β			−0.25			−0.01			−0.05	0.19	**0.43**	**0.74**	**0.69**	**0.94**	**1.13**	
	*p*			0.155			0.961			0.799	0.205	0.004**	<.001***	<.001***	<.001***	<.001***	
Physical	Mean	2.9	2.8		3.2	3.2		3.4	3.3		0.3	0.5	0.2	−0.1	0.5	0.5	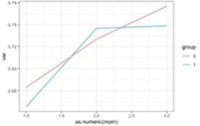
	SD	0.8	0.9		0.8	0.7		0.8	0.9		0.8	1.0	0.8	0.7	0.7	1.1	
	β			−0.10			0.14			−0.12	**0.29**	**0.53**	0.28	0.01	**0.57**	**0.54**	
	*p*			0.643			0.531			0.587	**0.068^**	0.001**	0.114	0.931	<.001***	0.001**	
Mental	Mean	3.9	3.9		4.1	4.2		4.3	4.1		0.1	0.4	0.2	−0.2	0.3	0.3	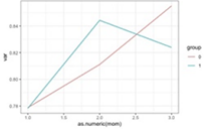
	SD	1.0	0.9		0.7	0.8		0.8	0.9		0.9	1.1	0.7	1.0	1.0	1.1	
	β			−0.12			0.36			−0.08	0.03	**0.51**	0.29	−0.15	0.32	**0.36**	
	*p*			0.612			0.156			0.770	0.891	0.013*	0.174	0.506	0.138	**0.086^**	
WIQ																	
WIQ distance	Mean	25.3	32.1		70.4	67.6		70.1	70.8		44.7	33.6	−4.67	−1.9	46.0	37.0	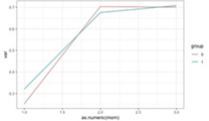
	SD	26.1	27.3		30.7	33.2		36.7	35.0		35.5	40.0	36.0	27.1	36.5	33.9	
	β			0.01			0.20			0.03	**1.31**	**1.50**	0.47	0.30	**1.78**	**1.80**	
	*p*			0.980			0.533			0.940	<.001***	<.001***	0.108	0.305	<.001***	<.001***	
WIQ speed	Mean	21.3	20.4		38.6	38.8		41.7	39.8		17.0	18.2	1.5	−2.1	21.6	18.7	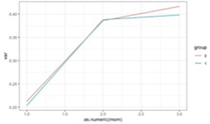
	SD	13.1	14.2		18.0	18.7		24.1	27.6		19.7	20.8	25.5	24.9	24.2	28.1	
	β			−0.11			0.03			−0.32	**0.82**	**0.96**	**0.38**	0.03	**1.20**	**0.99**	
	*p*			0.709			0.903			0.251	<.001***	<.001***	**0.087^**	0.895	<.001***	<.001***	
WIQ stairs	Mean	42.9	38.2		58.1	57.8		77.8	64.6		14.5	19.2	18.6	2.6	33.9	25.8	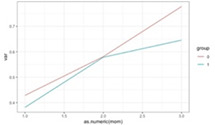
	SD	36.0	30.2		35.9	36.8		29.7	37.8		50.1	52.1	36.1	43.2	44.8	46.0	
	β			−0.08			0.12			−0.43	**0.76**	**0.96**	**0.74**	0.20	**1.50**	**1.16**	
	*p*			0.789			0.705			0.197	0.019*	0.002**	0.020*	0.543	<.001***	<.001***	
Treadmill walking																	
PFWD	Mean	150.8	95.6		292.7	254.5		320.0	295.3		143.4	158.9	13.6	41.2	165.3	202.5	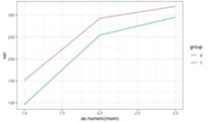
	SD	104.6	73.7		160.4	160.4		173.7	230.1		155.2	140.0	154.5	152.2	148.2	218.7	
	β			−55.14			−38.84			−18.45	**142.56**	**158.86**	21.42	41.81	**163.98**	**200.67**	
	*p*			0.119			0.291			0.635	<.001***	<.001***	0.459	0.153	<.001***	<.001***	
FWD	Mean	219.4	160.3		368.4	359.1		423.	373.7		149.4	201.1	37.1	10.3	195.1	219.0	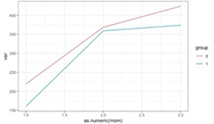
	SD	137.1	97.4		162.3	188.0		193.7	228.9		158.1	154.9	169.7	147.9	143.2	202.9	
	β			−59.08			−8.26			−39.72	**149.19**	**200.01**	45.71	14.25	**194.9**	**214.26**	
	*p*			0.127			0.837			0.349	<.001***	<.001***	0.116	0.632	<.001***	<.001***	
MWD	Mean	318.2	285.1		479.7	477.1		547.7	532.2		160.9	193.2	43.6	26.4	226.2	230.7	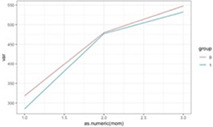
	SD	144.7	134.0		211.1	245.8		266.2	292.4		204.3	181.6	206.3	131.1	226.2	221.7	
	β			−0.15			0.05			0.04	**0.74**	**0.94**	**0.52**	**0.50**	**1.25**	**1.44**	
	*p*			0.649			0.863			0.899	0.001*	<.001**	0.017***	0.017*	<.001***	<.001***	
6-minute walking																	
6MWD	Mean	341.6	318.4		372.6	354.5		393.6	363.5		25.4	36.2	12.6	3.2	42.5	43.1	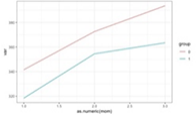
	SD	78.1	77.1		78.3	72.7		70.5	84.1		61.4	56.3	58.9	48.9	64.2	59.2	
	β			−23.15			−13.94			−24.10	**27.00**	**36.20**	15.84	5.68	**42.83**	**41.87**	
	*p*			0.198			0.446			0.200	0.006**	<.001***	0.124	0.582	<.001***	<.001***	
6MPM	Mean	2.5	2.4		3.1	3.0		3.4	3.2		0.7	0.7	0.3	0.2	0.8	0.7	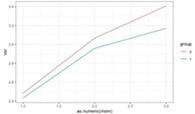
	SD	0.8	1.0		1.1	1.2		1.3	1.3		1.2	1.3	1.6	1.2	1.4	0.9	
	β			−0.03			−0.07			0.04	**0.45**	**0.40**	0.02	0.10	**0.43**	**0.50**	
	*p*			0.900			0.753			0.862	0.014*	0.025*	0.934	0.606	0.028*	0.007**	
6MStop	Mean	0.9	1.1		0.5	0.6		0.3	0.6		−0.5	−0.4	−0.2	−0.03	−0.6	−0.5	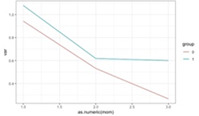
	SD	1.0	1.4		0.6	0.8		0.5	1.1		1.1	1.5	0.7	0.8	0.9	1.4	
	β			0.04			0.08			0.65	**−0.61**	**−0.58**	−0.57	0.002	**−1.19**	**−0.58**	
	*p*			0.877			0.832			0.138	0.018*	0.011*	0.146	0.993	0.001**	0.014*	

Means and graphs obtained with raw data; β is the model estimate and *p* is the *p*-value provided by the corresponding regression model. PFWD: Pain-free treadmill walking distance (in meters); FWD: Functional treadmill walking distance (in meters); MWD: Maximal treadmill walking distance (in meters); 6 MWD: 6-Minute walk distance (in meters); 6MPM: 6-Minute pain distance (in minutes); 6MStop – 6-Minute number of stops. Significance: ^*p *< 0.1, *p < 0.05, **p < 0.01, ***p < 0.001. Number of participants (n) may differ in some rows. Significant (or marginally significant) values are highlighted in bold. Red line, CG: blue line, EG.

Therefore, we have proceeded with further modeling analysis in order to understand whether the relationships of the primary outcomes with other variables have evolved differently for the two groups over time. First, we performed an internal analysis of the outcome to investigate how baseline scores evolved over time. Second, we conducted an external analysis to understand how other baseline variables affected the outcome as time went by. To perform the group comparison, several multilevel models with three-way interactions of the form “Variable x Group x Moment” have been evaluated. Interestingly, this modeling analysis only detected between-group differences for the MWD outcome. More specifically, unlike the control group, in the EG the MWD has never lost its association with the baseline scores over time (more than that, time strengthened this relationship). Moreover, patients of the EG with no extreme anxiety showed a higher increase in the MWD by the 6-month follow-up, compared with the CG. We emphasize that the sample size of this study only allowed to detect large effect sizes and therefore, all significant effects found in this study are strong. Details of these two models are given below.

### 7.4. Between-group differences in the MWD over time: (a) the predictive power of baseline MWD, and (b) the extreme anxiety diagnosis at the baseline

Although from the quantitative point of view, there is no significant between-group difference in the MWD improvement (score discounting the baseline MWD) over time, after controlling for participants' differences at the baseline, it is possible to uncover some important differences. Results are shown in Table 4. More specifically, the predictive power of participants' baseline MWD over time was significantly different for the two groups. In the CG, this predictive power did not vary significantly over time; however, in the EG, it increased and became significantly higher at T2 (estimate difference of 4.89, *p* = 0.013) and T3 (estimate difference of 4.67, *p* = 0.024, at T3), when compared to the CG. This means that in the EG, a part of the improvement is explained by participants' baseline MWD, in contrast to the CG, where this causal relationship was not observed. Thus, participants from the EG improved a proportion of their baseline MWD and, as a result, the higher the baseline MWD, the higher the improvement achieved afterward. This between-group difference is particularly prominent in the 6-month follow-up, with an improvement of 5.17 (*p* = 0.003) in the EG and 0.44 (*p* = 0.779) in the CG. Thus, the baseline MWD only predicts the MWD improvement at T3 in the EG. Notice that this is a qualitative between-groups difference in the MWD over time.

**Table 4:** Between-group differences in the MWD over time: (a) the predictive power of baseline MWD, and (b) the extreme anxiety diagnosis at the baseline.

Model	Predictor			T1			T2			T3			Within-group change								
													T2-T1			T3-T2			T3-T1		
				CG (*n* = 35)	EG (*n* = 38)	EG-CG (*n* = 73)	CG (*n* = 34)	EG (*n* = 34)	EG-CG (*n* = 68)	CG (*n* = 30)	EG (*n* = 30)	EG-CG (*n* = 60)	CG (*n* = 34)	EG (*n* = 34)	EG-CG (*n* = 68)	CG (*n* = 30)	EG (*n* = 30)	EG-CG (*n* = 60)	CG (*n* = 30)	EG (*n* = 30)	EG-CG (*n* = 60)
Model 1	MWD baseline		β	4.36	4.30	−0.06	2.17	7.07	4.89	4.80	9.47	4.67	−2.18	2.77	**4.95**	2.62	2.40	−0.22	0.44	5.17	**4.73**
			*p*	0.002**	0.003***	0.977	0.096^	< .001***	0.013*	< .001**	<.001***	0.024*	0.144	0.101	0.028*	0.079^	0.162	0.923	0.779	0.003**	0.044*
Model 2	Extreme anxiety baseline	No	β	−0.64	−0.98	0.34	−0.01	0.18	0.20	0.54	1.11	0.57	0.62	1.16	0.54	0.55	0.92	0.37	1.17	2.09	**0.91**
			*p*	0.022*	<.001***	0.395	0.961	0.526	0.618	0.047*	<.001***	0.154	0.019*	<.001***	0.180	0.031*	0.001**	0.331	<.001***	<.001***	0.026*
		Yes	β	−0.96	−0.79	0.16	0.03	−0.13	−0.15	0.46	−0.12	0.58	0.98	0.67	−0.32	0.43	0.00	−0.43	1.42	0.67	0.75
			*p*	0.018*	0.022*	0.758	0.947	0.695	0.764	0.238	0.708	0.253	0.011*	0.038*	0.528	0.259		0.378	<.001***	0.040*	0.137

Means and graphs obtained with raw data; β is the model estimate and *p* is the *p*-value provided by the corresponding beta regression model. T1: Baseline; Time 2: 3-month; Time 3: 6-month; PFWD: Pain-free treadmill walking distance (in meters); FWD: Functional treadmill walking distance (in meters); MWD: Maximal treadmill walking distance (in meters); 6 MWD: 6-Minute walk distance (in meters); 6MPM: 6-Minute pain distance (in minutes); 6MStop: 6-Minute number of stops. Significance: ^*p* < 0.1, *p < 0.05, **p < 0.01, ***p < 0.001. Number of participants (n) may differ in some rows. Only the significant values for the group difference on “within-group change” are in bold.

The predictive power of MWD was intensified over time, meaning that participants from the EG benefited from the WalkingPad app, except those with a weak walking ability (Figure 1). It also indicates that the MWD in the EG, unlike in the CG, exhibited strong stability over time.

**Figure 2:**
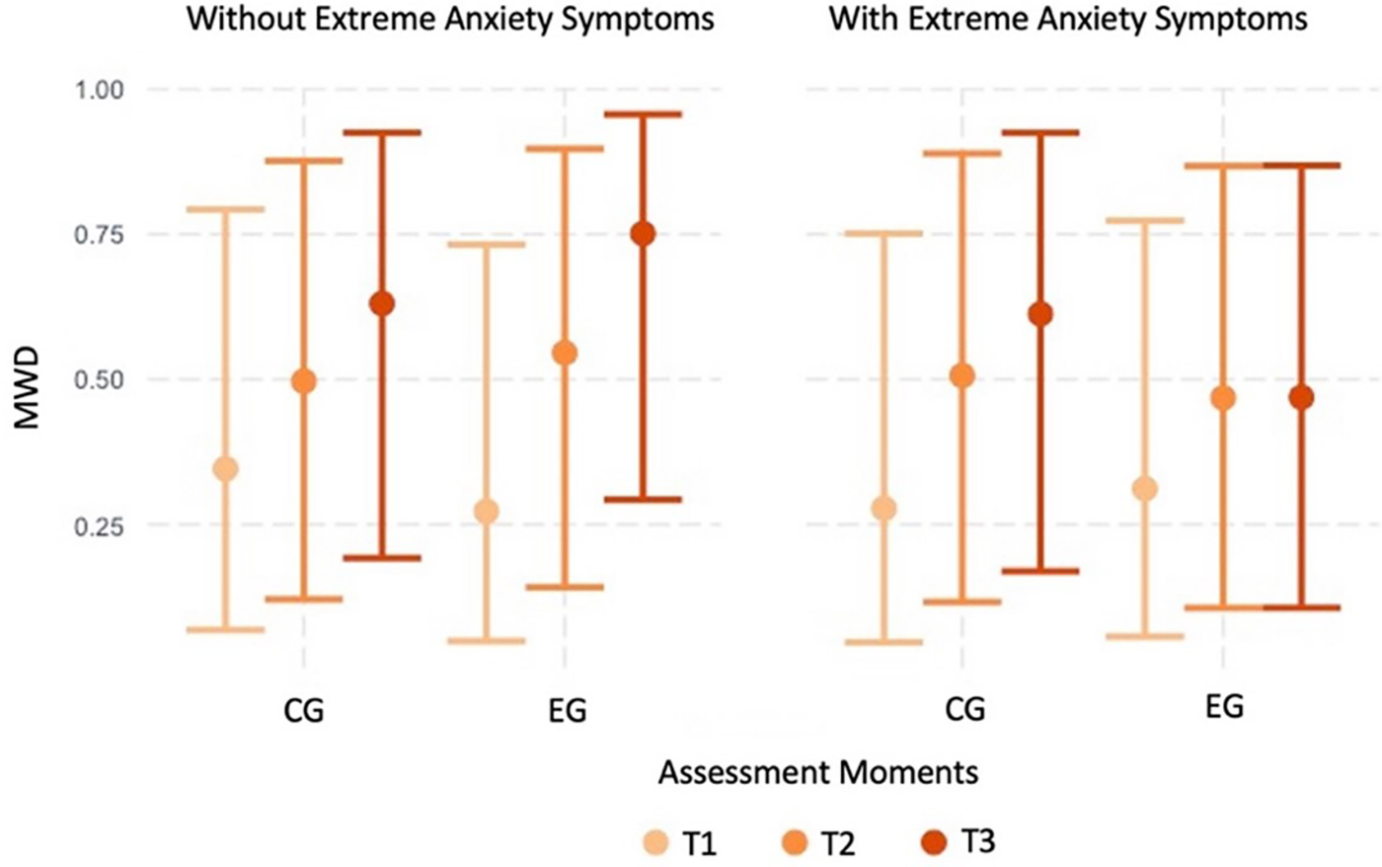
Graphic representation of the between-group differences in the average MWD over time (fitted data), for participants with and without extreme anxiety symptoms.

**Figure 3:**
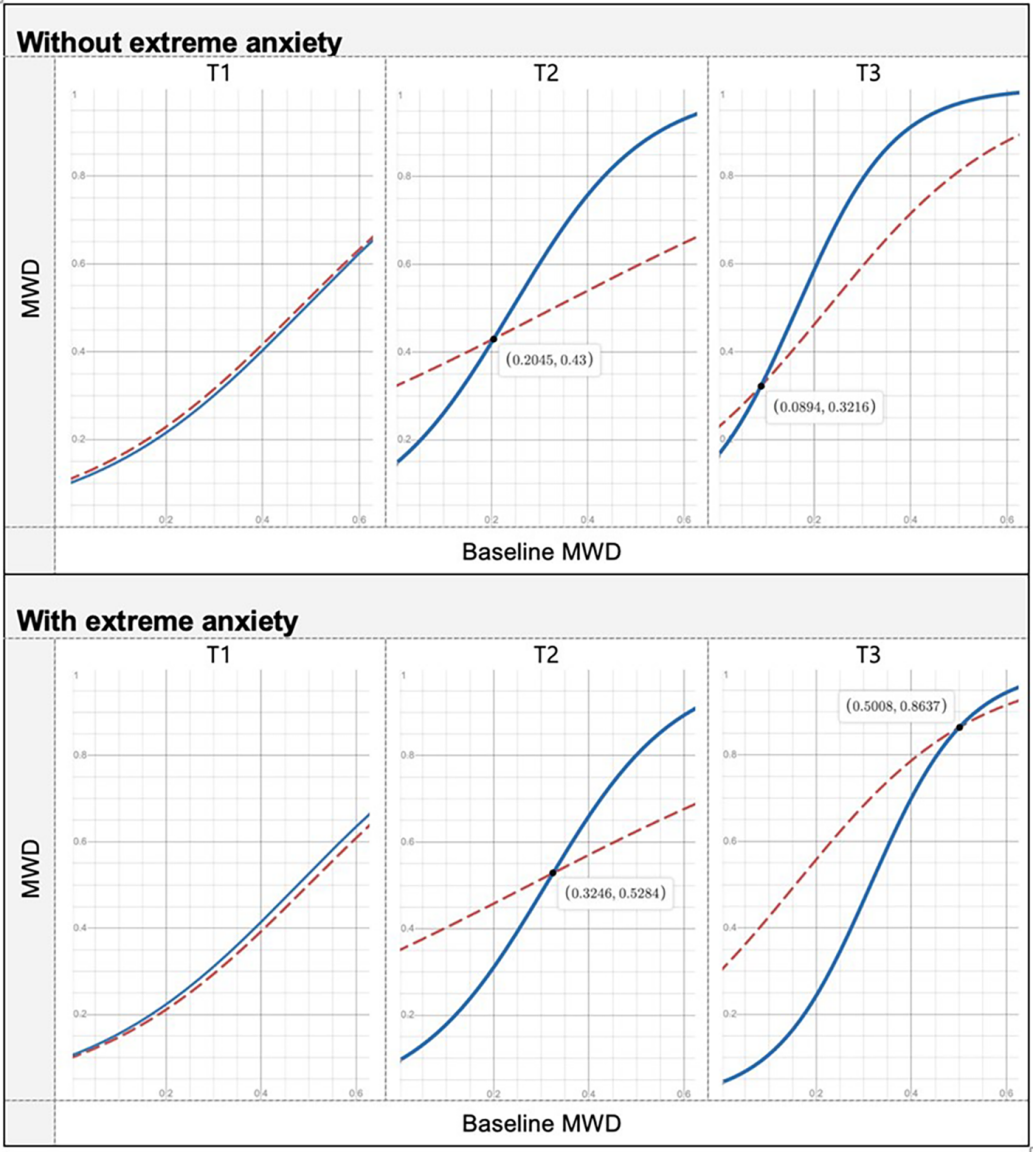
Graphic representation of the between-group differences in the average MWD over time (fitted data), at each time point, separating the cases with and without extreme anxiety. Red line, CG: blue line, EG.

Furthermore, anxiety (fixed at baseline) had a significant between-group different effect on MWD (depression also showed a similar result, but it disappeared when controlling for anxiety). Except for patients with extreme anxiety symptoms at the baseline, the EG showed a higher increase in the MWD at T3 (increase difference = 0.91, *p* = 0.026). For patients with extreme anxiety symptoms at the baseline, the increase from T1 to T3 was not significantly different for the two groups (increase difference = 0.75, *p* = 0.137) (Figures 2, 3).

## 8. Discussion

The WalkingPad program significantly improved MWD over time (from T1 to T2 and from T2 to T3) and PFWD and FWD from T1 to T2. If, on the one hand, improvements in distances are achieved essentially in the first 3 months, on the other hand, the other three months are essential to improve the MWD. Therefore, these results support the 6-month duration of an HBET. Furthermore, the results found for walking distances on treadmill evaluation were much higher than those found in other studies that also focused on the effects of an HBET supported by a psychological intervention based on behavioral change or education (53–56). However, only one study had a 6-month follow-up and did not achieve these promising results (55). The increase in 6 MWD was also significant from T1 to T2 (32 m), similar to other studies (1, 53–56) but not from T2 to T3, as expected (57, 58). These results may reflect the effectiveness of the behavior change intervention, carried out face-to-face and by telephone in the 6 months, in promoting a more integrated type of motivation and, consequently, greater adherence to physical exercise (10, 12, 23–28). The WalkingPad app was innovative as it effectively improved MWD at 6 months.

Walking distance and speed increased in the first 3 months but not in the last three. In turn, climbing stairs increased over time: from T1 to T2 and T2 to T3.

Disease-related QoL increased significantly over time, but general mental and physical QoL only increased from T1 to T2, suggesting that the effect of the WalkingPad program on disease-specific QoL was significant and progressive, reinforcing the advantage of a 6-month program to achieve improvements in quality of life. QoL of PAD patients is significantly associated with claudication pain, walking distance, and stairs climbing (7); thus, as walking distances and skills increase, so does QoL over the 6 months. General physical and mental QoL is subject to the influence of other external factors and only increases in the first 3 months, although it proves the effectiveness of physical exercise for the subjective perception of QoL.

Regarding the experimental group, the WalkingPad app positively affects patients' MWD, with higher baseline scores being associated with a stronger positive effect over time. However, this positive effect was not observed for people with weak walking ability and extreme anxiety symptomatology at the baseline. Therefore, this app may be recommended to people who score mid-to-high at the baseline MWD unless they have extreme anxiety symptomatology. For people scoring low at the MWD or having extreme anxiety symptomatology at the baseline, the app did not have a significant effect contrary to the expectations and results found in other studies (14, 16, 59, 60).

### 8.1. Clinical implications

The WalkingPad app may be recommended to PAD patients, except for those with weak walking ability and extreme anxiety symptoms. We hypothesized that the app can trigger more anxiety in people who are already anxious. Thus, in clinical practice, the health professional must assess the patient's anxiety to understand whether the physical exercise prescription should involve the app or not.

The 6-month duration is crucial to achieving results in maximal walking distance and QoL, as well as the behavioral change intervention that seems to have been the essential driver for promising results. Thus, this is the first study with a 6-month follow-up that achieved promising results in walking distances and quality of life. The behavior change intervention carried out in person and by telephone during the 6 months was decisive for the effectiveness of the physical exercise program, considering the importance of behavior change for the consistent adoption of a healthy habit. This study also shows that physical exercise programs should be at least 6 months in duration because disease-related QoL increased significantly over time, but overall mental and physical QoL only increased from T1 to T2, suggesting that the effect of WalkingPad program on QoL was progressive and not immediate. Furthermore, this study suggests that a smartphone app may not be useful for all patients. In fact, the app may be recommended for people with medium to high baseline MWD scores, unless they have extreme anxiety symptomatology, and for those who still have good walking skills (high MWD) at baseline, associated with a stronger positive effect over time.

### 8.2. Study limitations and futures studies

This study has some limitations that must be recognized. The sample was collected in only one hospital, being a unicentric study, and the results cannot be generalized to structured and supervised interventions (SET). Furthermore, the lack of gender heterogeneity in the sample (larger number of male patients) and the single-blind nature of the study, when the ideal was to be a double-blind study, must be recognized as limitations.

The sample size was small. Data were analysed according to the most recent statistical recommendations for longitudinal data, namely, using multilevel (or mixed-effect) models with suitable families of distributions. Several models have been investigated and we only report those (two) that were able to detect significant effects. Models are like microscopes whose augmenting power is defined by the sample size, with larger size providing greater power. We emphasize that the sample size of this study only allowed the detection of large effect sizes (increasing the sample would probably detect more effects), and therefore, all significant effects found in this study are strong. For this reason, we believe that our results will be easily corroborated by future studies, as long as they use the same conditions and the same statistical methodology (R codes used in this project can be provided on formal request).

The study lasted only 6 months, and the durability of the program's effects was unknown. Thus, more research is needed to determine the durability of these findings. An exploratory and usability study must be carried out to understand which application functionalities should be improved to promote the other results, and the same protocol should be applied, including more intense and personalized psychological follow-up for more anxious people. Furthermore, future studies should test the differences between gender in the use of the app and how this use influences adherence to physical exercise and outcomes.

## 9. Conclusions

A 6-month home exercise program supported by a behavior change intervention improved distances and walking skills as well as the physical, mental, and disease-related quality of life among adults with PAD and IC. The group that used the WalkingPad app improved their MWD in 6 months compared to the control group, except for patients with poor walking ability and extreme anxiety symptoms, which suggest the effectiveness of the WalkingPad app for patients with high walking ability and no severe anxiety symptoms.

## Data Availability

The raw data supporting the conclusions of this article will be made available by the authors, without undue reservation.
