# Supplementary material for: Effect of a monitored home-based exercise program combined with a behavior change intervention and a smartphone app on walking distances and quality of life in adults with peripheral arterial disease: the WalkingPad randomized clinical trial
Source: Front Cardiovasc Med. 2023 Nov 22;10:1272897. doi: 10.3389/fcvm.2023.1272897 (PMC10702743; doi:10.3389/fcvm.2023.1272897)
Supplement: Supplementary file 2 [file Datasheet2.pdf]

MANUAL DE INSTRUÇÕES

WalkingPAD

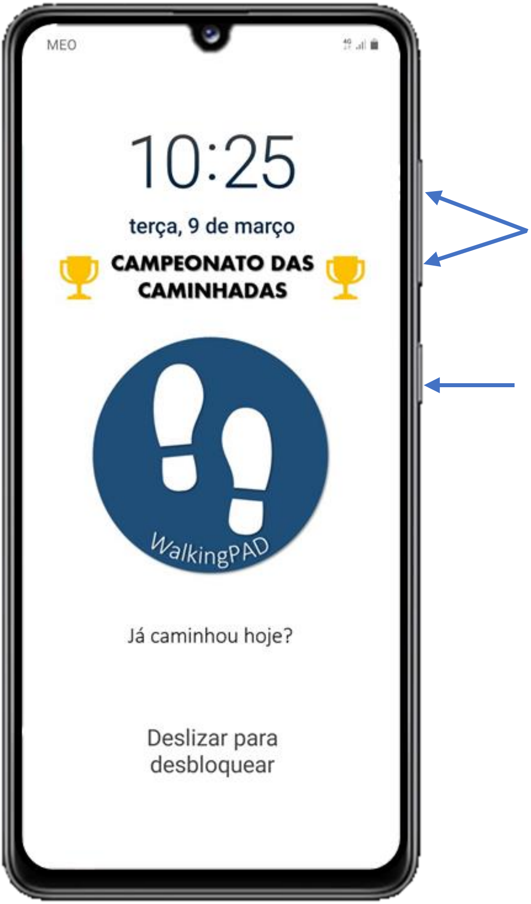

Botões para controlar o volume

Botão para ligar o ecrã

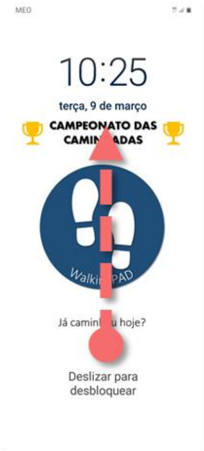

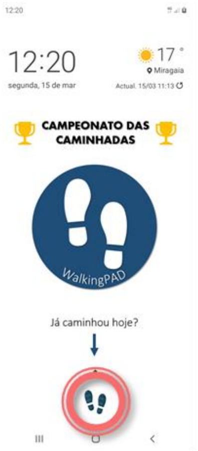

1

Para desbloquear o telemóvel arraste o dedo de baixo para cima no ecrã.

2

Para abrir a aplicação, toque no botão redondo localizado no fundo do ecrã que está assinalado na imagem acima.

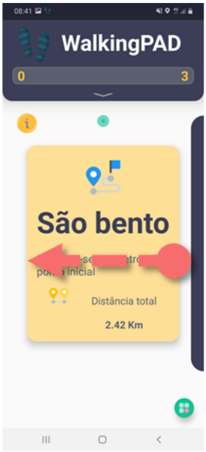

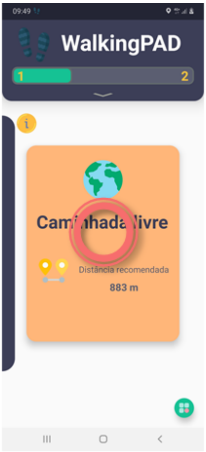

3

Para ver os seus percursos de caminhada arraste o dedo no ecrã da direita para a esquerda.

4

Para selecionar o percurso de caminhada toque com o dedo no cartão da caminhada pretendida.

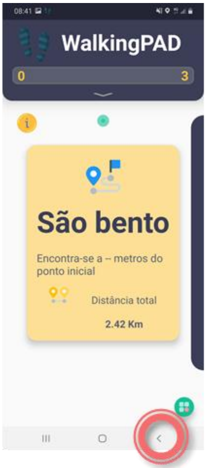

5

Se se enganar, poderá voltar atrás no botão assinalado.

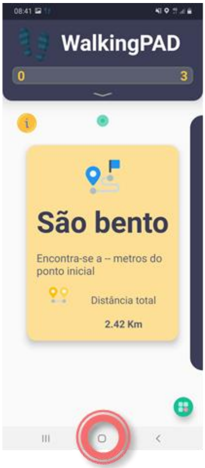

6

Para fechar a aplicação pode carregar no botão assinalado.

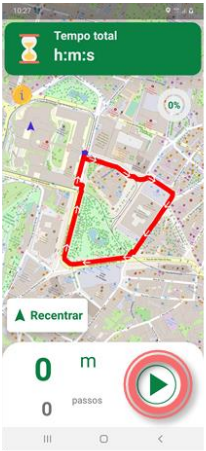

7

Para iniciar a caminhada carregue no botão verde assinalado na imagem acima.

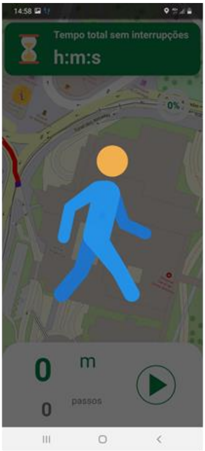

8

O telemóvel faz uma contagem decrescente e quando aparecer o bonequinho da imagem, inicie a sua caminhada!

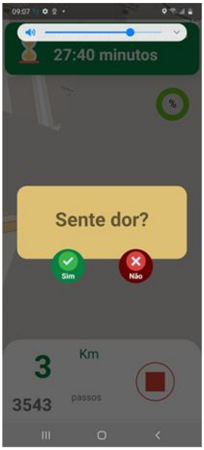

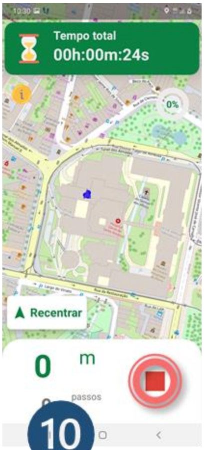

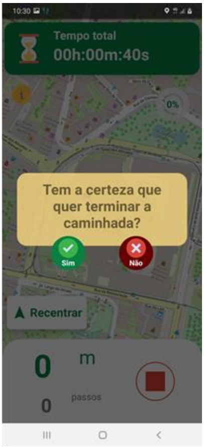

9

Sempre que parar de caminhar o telemóvel irá fazer um sinal sonoro e perguntar se parou porque sente dor. Se sim, carregue no botão verde, se não carregue no botão vermelho.

10

Caso queira terminar a caminhada antes de completar os 30 minutos recomendados, carregue no botão vermelho assinalado na imagem.

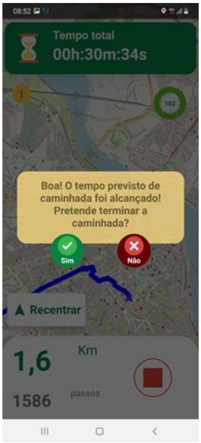

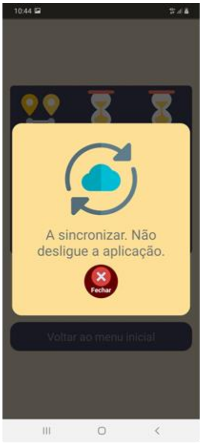

11

Quando completar os 30 minutos, o telemóvel emite um sinal sonoro e pergunta-lhe se quer continuar a sua caminhada. Responda SIM ou NÃO.

12

Quando terminar a sua caminhada aguarde que o telemóvel faça a sincronização.

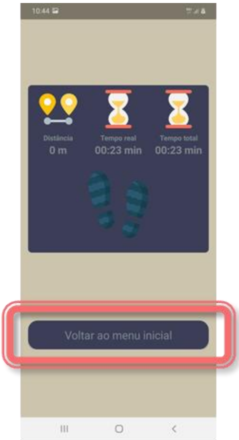

13

Carregue no local assinalado na imagem acima para voltar ao menu inicial.

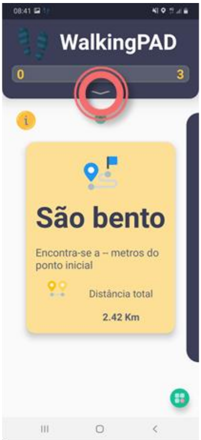

14

Poderá visualizar as suas caminhadas carregando na seta assinalada na imagem.

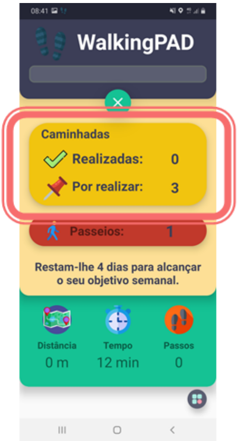

15

Pode ver quantas caminhadas faltam fazer para completar o objetivo semanal. Só serão contabilizadas para este objetivo, as caminhadas com duração superior a 30 minutos.

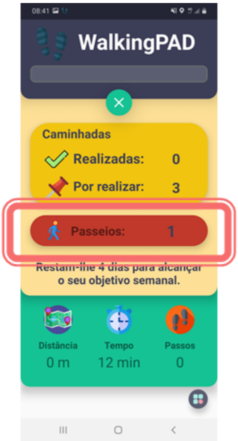

16

As suas caminhadas com duração inferior a 30 minutos são guardadas como passeios e pode consultá-las carregando no local indicado na imagem.

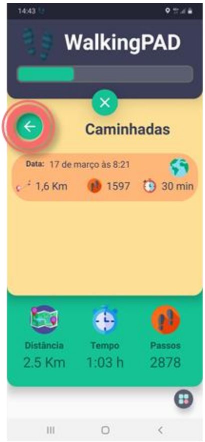

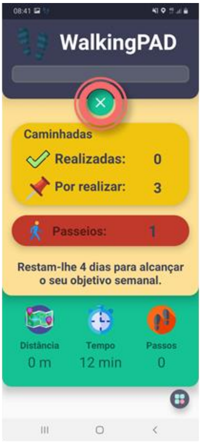

**17** Para voltar atrás carregue na seta assinalada na imagem acima.	**18** Para voltar ao menu inicial carregue na cruz assinalada na imagem acima

Em caso de dúvida contacte:

Apoio técnico ao utilizador WalkingPAD

969106369

Notas:

MANUAL DE INSTRUÇÕES

Projeto de Investigação

WalkingPAD

Março de 2021
