# Supplementary material for: Effect of a monitored home-based exercise program combined with a behavior change intervention and a smartphone app on walking distances and quality of life in adults with peripheral arterial disease: the WalkingPad randomized clinical trial
Source: Front Cardiovasc Med. 2023 Nov 22;10:1272897. doi: 10.3389/fcvm.2023.1272897 (PMC10702743; doi:10.3389/fcvm.2023.1272897)
Supplement: Supplementary file 3 [file Datasheet3.pdf]

Nome: ACSF **Processo:** 823652 **Grupo:** GE

GUIA DE INSTALAÇÃO DA APLICAÇÃO WALKINGPAD

Play Store

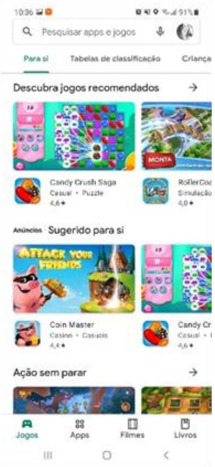

1. Abrir a aplicação ‘**Play Store**’

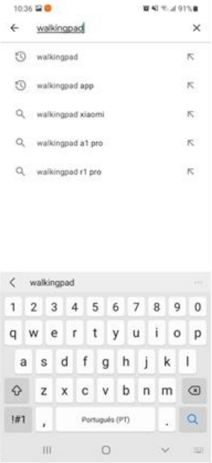

2. Pesquisar “Walkingpad”

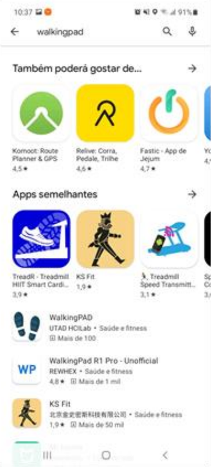

3. Carregar na aplicação ‘**WalkingPAD**’ (imagem dos pés)

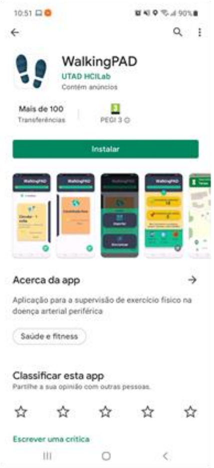

4. Carregar em “**Instalar**”

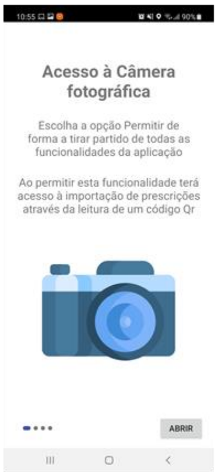

5. Abrir a aplicação WalkingPAD e carregar em “**Abrir**”> “**Permitir**”> “**Seguinte**” (repetir as vezes necessárias)

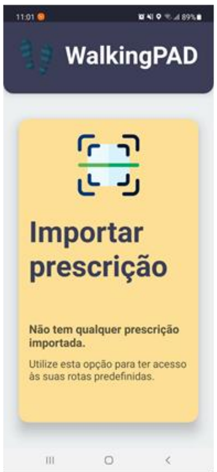

6. Carregar em “**Importar prescrição**” e pontar a câmara para o QRCode abaixo

QR code - Walking PAD

Processo: 307 Token: undefined

7. App está pronta a usar!
